# Housing Quality Improvement and Health Care Utilization: A Regression Discontinuity Study

**DOI:** 10.1002/pam.70074

**Published:** 2025-11-11

**Authors:** Kacie L. Dragan

**Affiliations:** 1 Department of Health Policy and Management, Columbia University Mailman School of Public Health, New York, New York, USA; 2 NYU Wagner Graduate School of Public Service, New York City, New York, USA

## Abstract

Low-income housing is often of substandard quality. Health care payers like Medicaid and Medicare are piloting reimbursement for housing quality improvement with the rationale of reducing expenses from acute care use, but little is known about whether housing quality interventions can alter health utilization. I evaluate a large-scale policy mandating remediation of NYC’s worst buildings in a regression discontinuity design to estimate the causal effect of housing improvements on health utilization. I do not find evidence of impacts on any metric of short-run acute care use or health expenditures, even among vulnerable subgroups. Exploratory longer-run event studies show some evidence of moderate (10%–15%) reductions in emergency department visits 3–4 years later but no evidence of impacts on expenditures. The policy succeeded at reducing housing violation rates in treated buildings by half. Yet, this only corresponded to a move from the 98th percentile to the 96th percentile of all buildings, underscoring the dire conditions tenants continue to live in—and suggesting one potential explanation for muted findings on health care use. The theory that housing quality interventions can directly translate to reductions in health use and spending may not be borne out empirically, particularly if interventions cannot completely address substandard conditions.

## 1 | Introduction

Low-income housing in cities across the United States is plagued by poor management, unsafe conditions, and the presence of potential health hazards like pests, lead, broken infrastructure, and mold. Recent estimates show that 42% of low-income households are living in housing requiring at least one major repair, and up to 20% of units in low-income neighborhoods have three or more serious quality issues (Divringi et al. 2019; NYC Department of Health and Mental Hygiene 2023; United States Government Accountability Office 2020). While the national housing affordability crisis has garnered urgent attention from policymakers and leaders across social service sectors, ensuring that the *quality* of housing available to low-income families is habitable, safe, and adequate poses a complementary—but often overlooked—challenge.

Qualitative accounts have documented the many ways in which living in poor-quality housing affects tenants’ quality of life—including low life satisfaction, feelings of hopelessness, powerlessness or failure, and stress and anxiety about the state of their living conditions—but most tenants have few alternatives to substandard rental housing other than homelessness (Garg et al. 2022; Marquez et al. 2019; Newton et al. 2022). Cross-sectional analyses in a range of contexts have found associations between low-quality housing and adverse health conditions, like asthma, lead poisoning, injuries, and poor mental health (Boch et al. 2020; Chakraborty et al. 2024; Keall et al. 2010; Krieger and Higgins 2002). Yet, making direct links between changes to housing quality and better health outcomes—and ultimately, lower health utilization—has proven difficult given the relatively sparse literature of controlled or natural experiments. Still, health sector actors like Medicaid managed care plans, state Medicaid programs via mechanisms like 1115 demonstrations, or Medicare Advantage plans have begun to explore health system-financed housing quality interventions, including using health insurance to reimburse for services like pest management or home modifications, with the explicit goal of decreasing short- to medium-run health expenditures from improved health (Enterprise Community Partners 2022; Hostetter and Klein 2020; Larson 2022; Livingston 2019; The Centers for Medicare and Medicaid Services 2024).

This paper leverages a large, multi-year housing quality improvement policy in a quasi-experimental regression discontinuity (RD) design to understand whether housing quality interventions affect health system utilization. In response to public concern about the alarming quality of low-income housing units, New York City (NYC) passed the NYC Safe Housing Law in 2007 (*Local Law No. 29 of 2007*), which authorized the Department of Housing Preservation and Development (HPD) to launch the Alternative Enforcement Program (AEP; NYC Department of Housing Preservation and Development 2022b). This law, now in its 18th year, requires HPD to identify the 250 lowest quality privately owned buildings in each year as ranked by an algorithm based on maintenance violations and emergency repair rates, and it mandates that building owners correct certain serious violations immediately. The ranking system and sharp cutoff generate an ideal comparison group of “untreated” buildings just under the selection threshold, allowing for estimation of the causal impact of intensive housing quality improvements on health care utilization of tenants in a regression discontinuity design.

In this evaluation of AEP, I find no evidence of changes in health expenditures or short-run utilization after the intensive housing quality remediation policy, despite a 50% reduction in total and severe violations in the treated buildings. In secondary event study analyses examining utilization up to 4 years later, there is moderate evidence of reductions in emergency department (ED) use but not in overall expenditures. The largely null results from this natural experiment are informative and contribute to the literature in several ways. This paper uses a strong causal inference design to provide evidence on whether housing quality improvement initiatives in extremely low-quality housing can move the needle on health utilization. While cross-sectional studies have identified a range of potential links between housing quality and injuries, mental health, and respiratory problems, the literature assessing links between actual *changes* to the home environment and lower health utilization has been sparse and mixed. The few randomized trials available have focused on highly specific interventions and populations (e.g., installing insulation, spraying for cockroaches).

By applying a strong causal design to a “real-world” policy, this paper sheds light on what decision makers in the health and housing sectors might realistically expect from housing quality improvement interventions. While there are many reasons to improve housing quality, the current rationale—especially in health sector policies like Medicaid 1115 waivers permitting reimbursement for services like housing remediation—has focused on the potential to reduce health system utilization or expenditures; in some public–private partnerships, expected short-run return on investment via reduced health system use has been an explicit condition of the programs (Enterprise Community Partners 2022; Larson 2022). The null findings in this evaluation are thus relevant for debates around how resources and responsibilities for housing quality are allocated across social service sectors. Finally, this analysis focuses on a highly vulnerable, though often overlooked population—low-income people living in privately owned housing—whose landlords are subject to less scrutiny than public or subsidized housing. Many prior cross-sector housing quality initiatives have focused solely on publicly owned housing, neglecting to consider the vast majority of low-income people living in privately managed apartments.

## 2 | Background

### 2.1 | Policy Context

Since its launch in 2007, AEP has generally been well-received by housing advocates and tenants and has been seen as one of the most powerful tools at the City’s disposal for holding private landlords accountable for the quality of the housing they offer. Many low-income families have few other housing options available to them, and fear of retaliation by landlords or a lack of awareness about habitability standards makes low-income and minoritized renters especially vulnerable to living in substandard conditions (Chisholm et al. 2020; Tilburg 2017). While NYC, like most municipalities, has had longstanding mechanisms in place for addressing the quality of housing through regular inspections of certain types of buildings and responses to tenant-initiated complaints, the NYC City Council designed AEP in 2007 to more systematically target the most persistently problematic buildings and enforce rapid improvements with real financial consequences for noncompliant landlords.

The program’s criteria and rules are written into the City’s charter and can only be modified by City Council vote. HPD’s process for ranking buildings has evolved over the program’s 18-year history per recommendations by HPD staff and City Council, but has always involved a calculation of the cumulative number of maintenance violations, amount of emergency repair charges the City has billed the building for, or a combination of the two (exact criteria by year are available in Appendix Table A1 in the Supporting Information). Violations are typically identified during HPD inspections following a complaint by a tenant, neighbor, city employee, or other concerned party. Depending on the policy’s rules in a given year, the number of buildings selected is either 200 or 250, and the lookback period for calculating rank is between 2 and 5 years. The frequent changes to the criteria by City Council vote make it challenging for landlords to perfectly replicate the algorithm themselves to predict their rank or “game the system” to avoid selection. Only privately owned buildings are subject to AEP since public housing managed by the New York City Housing Authority (NYCHA) has its own internal quality control programs.

In January of a given year, HPD notifies landlords of their selection into the program and requires that they remediate all heat or hot water violations, as well as 80% of serious mold, pest, rodent, and other “immediately hazardous” violations (e.g., peeling lead paint, broken fixtures or windows, water leaks, etc.) within 4 months of notification. HPD monitors progress with in-person visits and requires that landlords post notices informing tenants of the building’s participation in the program. Tenants are generally not relocated during the remediation process unless unavoidable. In an effort to increase the effectiveness of the program, HPD will offer low-interest loans to owners who cannot afford to remedy all issues within the first 4 months. Continued non-compliance results in liens and fees (particularly in cases where HPD hires contractors to make necessary emergency repairs after the 4-month compliance window). Through 2022, HPD had imposed $21.5 million in fees to noncompliant landlords and recouped $100 million for emergency repair costs they fronted to make buildings habitable for tenants (NYC Department of Housing Preservation and Development 2022a).

Reports published by HPD have shown notable improvements in these buildings, with a 50%–70% reduction in open mold or pest violations 4 years later (NYC Department of Housing Preservation and Development 2018, 2020, 2022a). Compliance and discharge rates are relatively high—particularly considering the buildings are typically run by NYC’s most notoriously negligent landlords—with roughly 60% of selected buildings being discharged within 12 months (Ashford 2018; NYC Department of Housing Preservation and Development 2018). While AEP does seem to motivate a large share of landlords into quality improvement and compliance, a challenge of the program has been long-run persistence of improvements: Of the 3137 buildings selected for the program from 2007 to 2022, 396 (13%) ended up back in the program in a subsequent cycle after being initially discharged.

### 2.2 | Prior Evidence and Theory

The theory underlying health sector programs to improve housing quality centers on the potential physical and mental health benefits of living in cleaner, safer, and more pleasant housing—and, subsequently, the potential for decreased health care utilization. Specifically, prior literature has established associations between mold or pests and respiratory conditions; unsafe housing and risk of injury; inadequate heating or cooling and heart and lung conditions; and low-quality living environments and poor mental health (Adamkiewicz et al. 2014; Beck et al. 2014; Boch et al. 2020; Chakraborty et al. 2024; Ellen et al. 2020; Glied 2020; Keall et al. 2010). However, nearly all of this literature has been purely cross-sectional, reporting point-in-time associations between housing quality problems and health problems, which makes disentangling correlation from causation or eliminating reverse causality challenging. Even if low-quality housing causally impacts health outcomes or health system utilization, people with pre-existing health challenges are also more likely to move into disadvantaged neighborhoods and housing, introducing the possibility that sicker individuals are “sorting” into lower-quality housing (Arcaya et al. 2014, Arcaya et al. 2016; Rolheiser et al. 2022; Ruel et al. 2010). Due to the cross-sectional nature of the literature, little is known about how long it takes for people living in substandard housing to develop health conditions—or how those conditions translate to actual health system utilization rates. Together, these limitations make it challenging to predict whether a program like AEP—or health sector policies targeting housing quality—can induce changes in health system utilization from improved health.

Some quasi-experimental studies have aimed to quantify the impact of housing quality interventions on health utilization, including non-controlled pre-post analyses, matching and difference-in-differences analyses, or instrumental variable analyses (Aizer et al. 2018; Ellen et al. 2020; Fyfe et al. 2020; Kersten et al. 2014; Sweet et al. 2014; Takaro et al. 2011). This body of work has generally found small but positive impacts of housing quality improvements on health outcomes, health system utilization, and other quality-of-life outcomes (e.g., education) in the short to medium term (1–3 years), suggesting the potential for housing remediation policies to have an impact on health utilization in some settings and groups, like children (Howden-Chapman et al. 2023). However, the identifying assumptions of these methods are difficult to empirically test and may be impacted by omitted confounding variables or regression to the mean, particularly in the pre–post designs. Additionally, several of these impact evaluations have focused on interventions or populations that are not generalizable to the broader low-income renter population that health system programs tend to target. For example, prior work has focused only on people in public housing (Ellen et al. 2020; Kersten et al. 2014; Takaro et al. 2011), has entailed complete redevelopment of low-income housing rather than remediation or renovation (Kersten et al. 2014; Takaro et al. 2011), or has been coupled with strong health education components, making impacts from the remediation activities difficult to disentangle from health education impacts (Sweet et al. 2014).

Several high-quality, though small, randomized controlled trials have looked at very specific types of remediation with mixed findings. Generally, these trials have identified improvements in air quality, self-reported health, or self-reported symptoms but have found weak or no evidence for changes in clinical metrics like health system utilization or results of lab tests. For example, Olson et al. (2021) conducted a randomized controlled trial of an integrated pest management intervention in NYC focused on children with asthma but found no measurable changes in health care utilization despite improvements in self-reported symptom rates. Randomized trials using air filters, pest management, improved ventilation, or mold remediation have found improvements in air quality but no detectable improvements in clinical measures of breathing ease or health system utilization (Barton et al. 2007; Burr et al. 2007; Eggleston et al. 2005). In New Zealand, Howden-Chapman and coauthors (2007) randomized an insulation improvement intervention in low-income housing, again finding improvements in self-reported symptoms but smaller or insignificant changes in health system utilization. While lead exposure has well-established links to neurological damage, even randomized trials of lead abatement interventions have failed to show meaningful changes in clinical measures of blood lead concentrations or developmental improvements in children (Braun et al. 2018).

Many of these null results may be, in part, attributable to the long and complex pathway from exposure, to symptoms, to actual health system utilization—and the associated challenges of intervening at the right time, to the right degree, among the right people. Low-income families often face many concurrent risks and have been exposed to substandard conditions for many years. Low-income, minoritized populations often have little control over the quality of their housing or alternatives to low-quality rentals, in part due to factors like structural racism and residential segregation (Marquez et al. 2019). These factors may limit the ability of any single housing quality intervention effort to influence health enough to alter utilization, even if self-rated health or subjective wellbeing improves. For example, in-home air quality improvements may have a limited impact on acute care use if low-income families are also living in neighborhoods with poor outdoor air quality and work or go to school in places with respiratory hazards (Tessum et al. 2021). Even if housing quality interventions improve wellbeing in other ways, which many of these studies suggest they do (e.g., self-reported health), understanding whether broad, intensive quality improvement policies like AEP are likely to have measurable impacts on health utilization or expenditures is important for informing the best possible policies in this area and for accurately framing their value, particularly as health sector payers like Medicaid and Medicare begin piloting reimbursement policies for housing quality remediation.

## 3 | Methods

### 3.1 | Data and Sample

The data for this study include individual-level records from Medicaid eligibility, claims, and encounters data in New York City from 2007 through 2019. Enrollees were assigned to their building of residence using their address on record at the beginning of a given AEP program year (typically mid- or late January). Addresses are maintained, verified, and updated regularly at re-enrollment intervals and during interactions between the social service system and enrollees in a centralized data warehouse and are generally of high accuracy (Ellen et al. 2020; The Coverage Learning Collaborative 2021). Any enrollee with at least 10 out of 12 months of enrollment in the given AEP program year was included in the analytic sample. The data include all inpatient, outpatient, and pharmacy claims covered by Medicaid. Enrollee-reported demographic information (date of birth, race/ethnicity, sex) is also available in enrollment records.

The rankings of all NYC buildings under consideration for AEP in every year of the program, as well as the underlying violation and emergency repair charge data HPD used to calculate rank, were obtained directly from HPD via a Freedom of Information Law (FOIL) request. Obtaining an exact rank from HPD was essential for the regression discontinuity design, as the design is estimating the local average treatment effect (LATE) of the policy exactly at the 200- or 250-building cutoff point; misclassifying buildings on either side would threaten the validity of the design. Additional data on building characteristics (building age, number of units, etc.) were obtained from the NYC PLUTO dataset linked using the borough-block-lot (BBL) identifier (NYC Department of City Planning 2022). AEP program years 2013, 2014, and 2015 were excluded from the analysis due to unavailable ranking data, and program year 2010 does not exist due to a change in the cycle timing from a November start to a January start (i.e., program year 2009 began in November 2009, but then the policy changed to a January start, requiring HPD to wait 14 months until January 2011 to issue the next AEP selections). Therefore, program years 2007, 2008, 2009, 2011, 2012, 2016, 2017, and 2018 were available for analysis.

### 3.2 | Exposure and Outcomes

I measured exposure to the AEP program using the rankings obtained from HPD. Ranks (or the “running variable”) were recentered such that the building ranked 0 was the first building over the selection threshold, and the building ranked 1 was the building that just missed the selection threshold. In all years, both emergency charge data and maintenance violation data were used by HPD to screen for the initial set of buildings eligible for inclusion, creating a universe of buildings up for consideration each year. In the years 2007–2009 and years 2016–2018, the set of eligible buildings was ranked by a violations-based metric, while in years 2011–2015, buildings were ranked using an emergency charges metric. All Medicaid enrollees living in the building were given the HPD rank corresponding to the building they lived in at the start of the AEP year, and results were pooled across years, such that each “rank” had many individual enrollees associated with it.

To demonstrate that the rankings were accurately capturing the treatment status of the buildings and their tenants, I ran the main regression discontinuity model (described below) using an indicator of AEP selection as the outcome, in which we would expect the probability of selection to go from 0% to 100% if the rankings provided by HPD were correct. Additionally, I ran the model using the number of open violations per unit as of 1 year after selection as the outcome (per publicly available violations data from 2012 to 2018) to confirm that AEP selection generated a sharp decrease in open violations at the cutoff. Seeing a discontinuity in the number of violations would indicate that the program had its intended effect of reducing exposure to housing quality issues.

The primary outcomes of interest were measures of acute health system utilization from the Medicaid claims data in the 12 months after a building’s initial selection into AEP. Acute utilization was chosen to pick up immediate impacts in the severity of or exacerbation of health conditions. I calculated the total number of ED visits, probability of one or more ED visits, and number of ED visits for a set of diagnoses likely to be related to housing exposures per prior literature (injuries, respiratory conditions, or anxiety; Ellen et al. 2020). I also measured a summed index of housing-sensitive visits, which could take a value of 0 to 3, to assess how many housing-sensitive conditions the individual was seen for in any setting (all inpatient or outpatient claims). As a global measure of utilization, I also used total expenditures. As secondary outcomes, I measured disenrollment from Medicaid by the second year after program start (to assess whether the treatment induced changes in who was observed), as well as the probability of moving out of the building by the end of the first year to assess whether improvements made people more likely to stay. The design of the AEP program is such that many buildings that are just under the cutoff in year *T* end up being above the cutoff in year *T* + 1. Around 30% of the control buildings in a given year are therefore only “untreated” for 1 year before they themselves are mandated to remediate quality issues under AEP. From a policymaking perspective, many current cross-sector housing quality improvement programs are designed with short-run (1–2 year) returns on investment in mind (Enterprise Community Partners 2022; Larson 2022; The Centers for Medicare and Medicaid Services 2024). For these reasons, all primary outcomes are measured for just a single year after AEP selection. In exploratory analyses, I extend the follow-up period up to 4 years later.

### 3.3 | Statistical Analysis

I leveraged a regression discontinuity design to estimate the LATE of the housing remediation policy. The RD design requires that a policy or program be implemented at a certain cutoff score of a continuous “running variable,” with the causal effect recovered under the assumption that units just below the threshold do not differ in observed or unobserved ways from the units just above the threshold (along dimensions that could confound the exposure-outcome relationship). In other words, the identifying assumption is that the cutoff is arbitrary and thus is “as good as random” for assigning units to the treated or untreated groups, allowing an unbiased, internally valid effect at the immediate region of the cutoff to be estimated (Chaplin et al. 2018). In the AEP setting, the running variable is the building’s rank per HPD’s data, and the cutoff is the 200th or 250th slot, depending on the program year (see Appendix Table A1 in the Supporting Information). Given the relatively arbitrary nature of the AEP ranking system and cutoff threshold, there is no reason to believe that units below the cutoff would systematically differ from those just above the cutoff. To provide evidence that the AEP program satisfies this assumption, I tested whether tenants in buildings on either side of the cutoff were similar across the cutoff in potential confounding characteristics (age, sex, baseline health spending, race/ethnicity, baseline Medicaid enrollment) and whether the buildings themselves were similar across the cutoff (borough distribution, size). For the regression discontinuity method to be valid, potential confounding characteristics should be smooth across the cutoff.

The jump in the outcomes at the threshold (the “discontinuity”) provides an estimate of the program’s causal effect. In line with best practices in RD studies (Cattaneo et al. 2019), I use local linear regression for observations within 25 ranks of the cutoff with a triangular kernel for weighting, in which observations closer to the cutoff are weighted to a greater degree than observations far from the cutoff. The cutoff of 25 ranks was chosen using a data-driven optimal bandwidth selection algorithm built by Cattaneo et al. (2019).

Computationally, the RD is essentially estimating a linear function on either side of the cutoff among observations within just a narrow (“local”) bandwidth—just 25 ranks—with the observations right around Rank 0 having slightly more weight than those farther from the cutoff. This can be thought of as a segmented regression in the immediate neighborhood of the cutoff (−25 < rank < 25) with the equation being represented by:

$$y_{ibt}=\beta_{0}+\beta_{1}\;{\text{RankOverThreshold}}_{ibt}+\beta_{2}{\text{Rank}}_{ibt}+\beta_{3}{\text{Rank}}_{ibt}*{\text{RankOverThreshold}}_{ibt}+\delta_{it}+\gamma_{bt}+\varepsilon_{ibt}$$

where *β*_1_ is our coefficient of interest, which captures the difference in outcomes at the cutoff in the year after AEP selection for enrollee *i* in building *b* at time *t*. *δ*_*it*_ and *γ*_*bt*_ represent individual and building-level characteristics (age, baseline health, sex, building size, borough, program year as a categorical variable, race/ethnicity), respectively. These covariates are included for the purpose of increasing precision of the estimates by reducing variance. Standard errors are robust, corrected for misspecification bias, and clustered at the building level (Cattaneo et al. 2019). As some degree of misspecification of the underlying functional form at the cutoff is expected in an RD design, Cattaneo et al. (2019) bias-corrected robust standard errors account for uncertainty that remains in the point estimate (“jump”) that is derived from a given bandwidth, even in large samples.

A series of secondary analyses testing sensitivity to RD assumptions (e.g., bandwidth and functional form) and heterogeneity among vulnerable subgroups (e.g., children, people with chronic conditions) are described in more detail in the Results. An additional exploratory analysis using an event study approach is also described, which enables examination of longer-run outcomes up to 4 years following the AEP intervention.

## 4 | Results

### 4.1 | Descriptive Statistics and Tests of Assumptions

The sample included eight AEP program years (2007–2009, 2011–2012, and 2016–2018; see Methods for more detail). In total, 48,151 Medicaid enrollees were matched to buildings in the list of low-quality buildings under consideration for AEP as of the start of an AEP year. Of these, 24,294 enrollees were within the 25-rank empirically driven regression discontinuity bandwidth, which included 14,974 untreated individuals (−25 < building rank < 0) and 9320 treated individuals (0 ≤ building rank < 25). The mean age of these enrollees was 26.28 years (SD: 21.10; median: 19.84; IQR: 8.75–41.70). The buildings selected for AEP were largely located in Manhattan, Brooklyn, and the Bronx, with these three boroughs (i.e., counties) making up 97.1% of the sample, due to the increased likelihood of buildings meeting the initial AEP screening criteria in these boroughs. (Older and larger multifamily housing is concentrated in these boroughs, as well as low-income housing generally, which is most likely to have quality issues.)

Notably, buildings selected for AEP had a baseline total violations rate (violations per residential units) in the 98th percentile of all multifamily buildings in NYC (*n* = 171,181). For example, as of December 2016, AEP buildings had a mean of 14.2 currently open violations per unit before AEP selection, while multifamily buildings in NYC overall had a mean of just 1.5 violations per unit. After AEP, buildings had a mean of 7.3 open violations per unit: half the baseline rate, but a value still in the 96th percentile of all buildings (or, in the 94th percentile of the 70% of buildings with at least one open violation). Even after targeted improvements, elimination of half of the open violations, and increased scrutiny and risk of fines to landlords, the average tenant in an AEP building was still in one of the very worst buildings in NYC. This statistic underscores how challenging the situation in these buildings is, even if the program is making substantial progress in improving quality.

**FIGURE 1 |:**
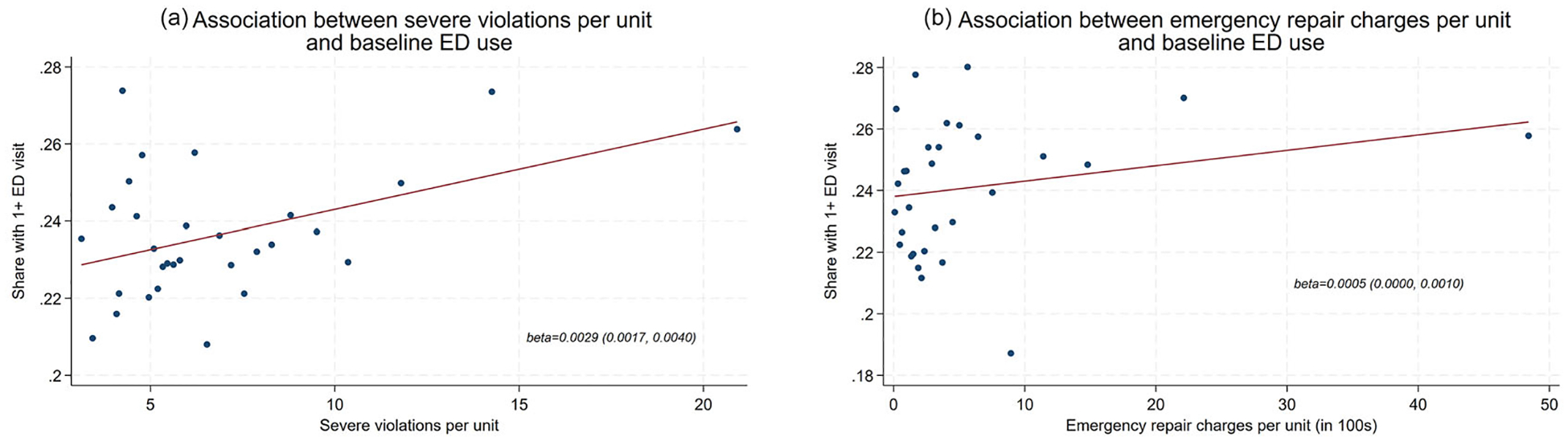
Cross-sectional correlation between housing quality and Emergency Department use

Understanding whether maintenance violations in general correlate with health system utilization in this population is important for contextualizing the study. In cross-sectional analyses of data from the years prior to AEP selection (or the near-miss year, for control buildings), both the violations rate and the emergency repair charges were positively correlated with acute care use. Each additional maintenance violation per residential unit was associated with a 0.29 percentage point higher probability of ED visits (95% CI: 0.17, 0.40), and each $100 increase in emergency repair charges per unit was associated with a 0.05 percentage point higher probability of ED visits (95% CI: 0.00, 0.10; Figure 1). These raw correlations are somewhat small (and are calculated only among the subset of NYC renters in the eligibility window for AEP), but this finding generally aligns with prior cross-sectional literature documenting an association between housing quality and worse health. This captures the underlying theory motivating a policy like AEP: that remedying the housing quality issues could plausibly improve tenant health and reduce acute care use, given that the two tend to track together. At the same time, the modest size of the correlation raises the possibility that housing quality (and thus, housing quality *improvements*) may not relate to utilization as strongly as previously hypothesized.

**FIGURE 2 |:**
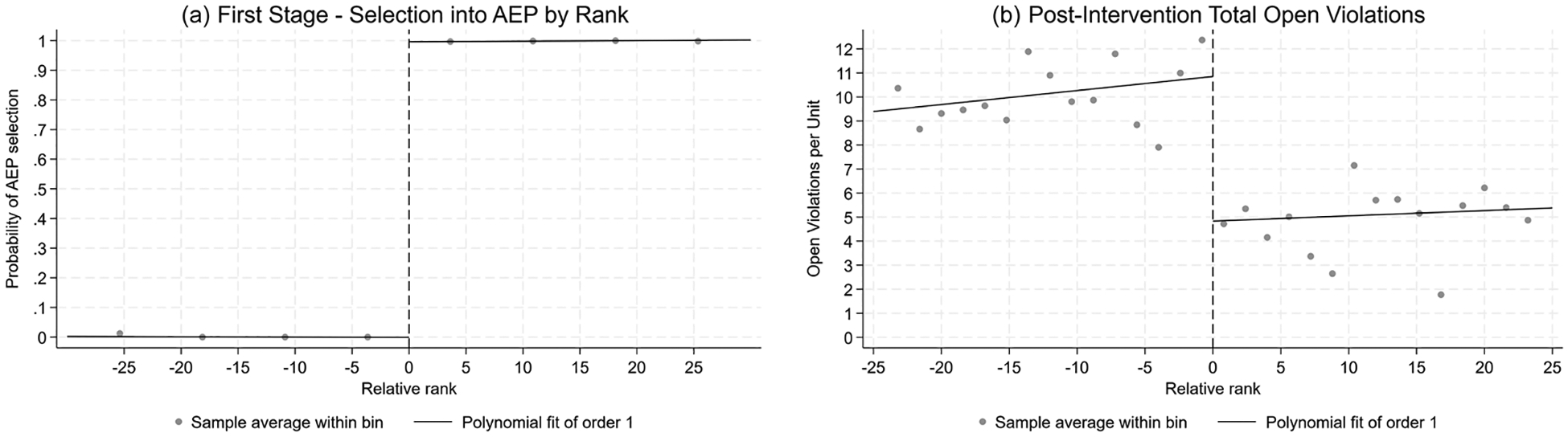
First-stage outcomes at Alternative Enforcement Program (AEP) treatment cutoff.

The ranking data provided by HPD through a FOIL request accurately corresponded to the probability of being in AEP (per the publicly available AEP list): probability of selection into AEP went from 0% to 100% at the relative rank threshold of 0, indicating a sharp and complete discontinuity (Figure 2a). Likewise, the number of open violations per unit as of 1 year post-selection decreased discontinuously at the threshold, decreasing by −6.01 violations per unit (95% CI: −9.25, −2.70) from a value of 10.85 just before the cutoff to 4.84 just after it (Figure 2b). For the most severe violations (class B and C only), the rate of open violations decreased by 4.50 from 8.01 violations per unit. These decreases by over 50% for both total and severe violations indicate that the program was effective, at least in the short run, at reducing the number of total maintenance problems tenants were exposed to. It also indicates that corrected violations were not merely “low-hanging fruit” that would not be expected to impact tenant wellbeing, but included the most urgent and severe issues as well. By 2, 3, and 4 years after initial AEP selection, the difference between the number of open violations in treated and control buildings (limited to control buildings not treated in subsequent years) shrinks to −2.78, −1.55, and −1.04 violations per unit, respectively (non-significantly different from zero; 95% CIs: [−6.43, 4.43], [−2.68, 7.00], and [−4.96, 6.19]). This suggests that the impact on housing quality may fade to some degree over time.

One risk of using the RD approach to assess the AEP program could be the threat of spillovers of the treatment onto the untreated buildings just below the threshold, particularly if landlords own multiple buildings on either side of the cutoff or if they notice other buildings in the neighborhood being entered into the AEP program. These landlords may initiate improvements or remediate violations on their own. To assess the threat of spillovers like these, I calculated the share of control buildings in the RD analytic bandwidth that were owned by a landlord that also had a building selected for AEP or were in the same census block as a treated building. A very small share of control buildings would have also been “exposed” to AEP either through the same landlord (2%) or block (7%). While landlord data are imperfect (given the use of LLCs and subsidiaries for rental ownership), these statistics suggest that very few, if any, control buildings are likely to have been induced into costly and extensive remediation on their own accord.

**FIGURE 3 |:**
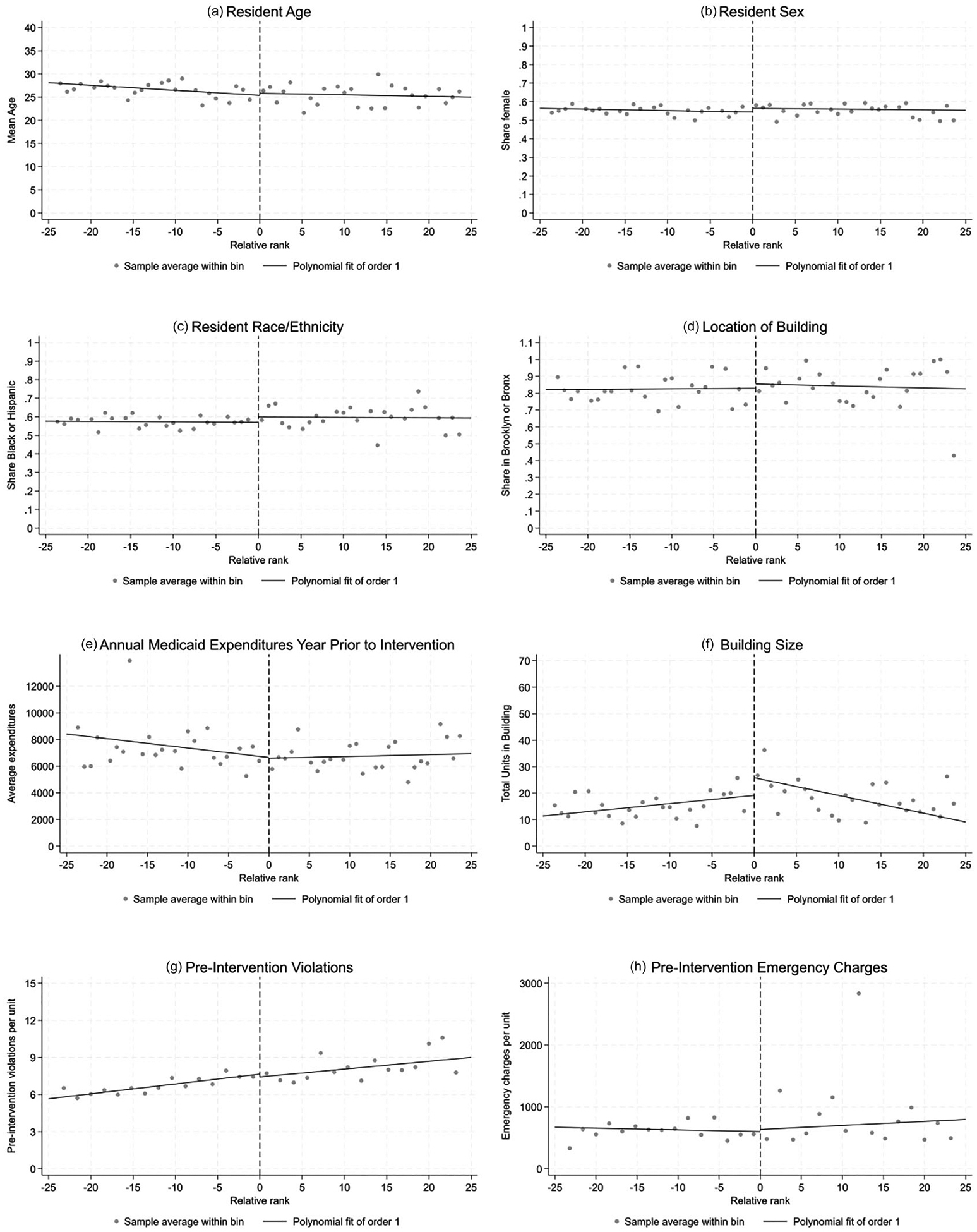
Covariate smoothness at AEP treatment cutoff.

I tested for the smoothness of a range of potential confounding characteristics across the cutoff threshold to build the case that this setting was appropriate for RD. Seeing no discontinuities in covariates (e.g., demographics) at the cutoff is consistent with the identifying assumption in the RD approach that the cutoff may be “as good as random” for assigning people to treatment or control status. Figure 3, Table 1, and Appendix Figure A1 in the Supporting Information summarize the findings from these tests, demonstrating that there are no large discontinuities in any of the covariates. None of the local linear regressions using the potential confounding variables as outcomes resulted in a significant jump at the AEP threshold, which can be seen visually in the figures and in the non-significant coefficient estimates. Additionally, the rates of severe violations and emergency charges (the two main AEP selection criteria) in the pre-intervention year are smooth at the cutoff. This provides evidence that the data and policy setting likely satisfy the assumptions for an RD analysis that the treatment assignment is as good as random. All RD analyses of the health utilization outcomes described below adjust for these covariates for the purpose of reducing variance and increasing precision of the estimates, even though these characteristics are balanced across the cutoff (i.e., are not confounders).

### 4.2 | Main Results

**FIGURE 4 |:**
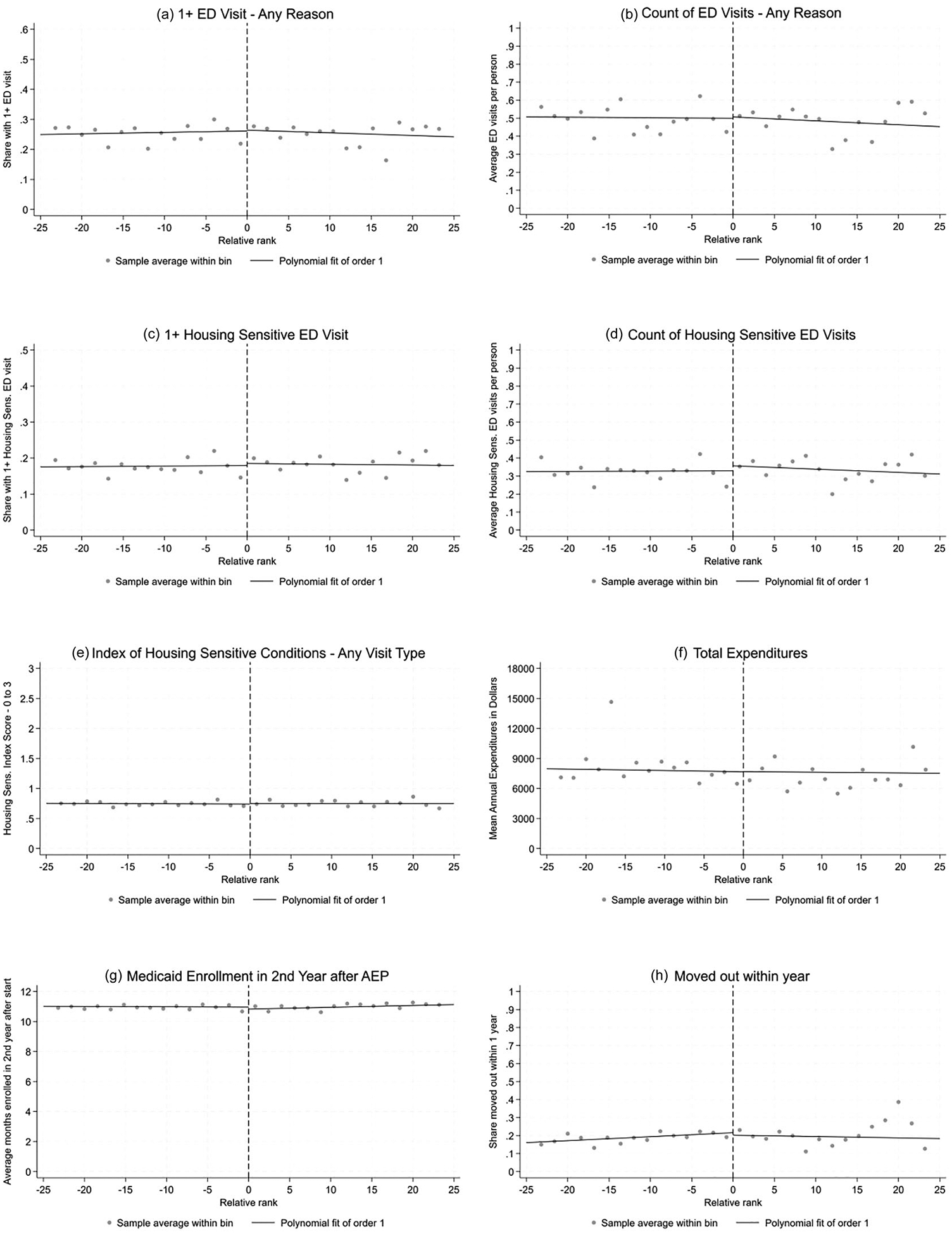
Regression discontinuity (local linear regression) plots for all outcomes in the year after intervention.

A summary of the core regression discontinuity results for all of the main outcomes is available in Table 2 and Figure 4. The main outcome of interest was acute care utilization as measured by ED visits, as acute care utilization rates can pick up short-run changes in severity of or exacerbations of existing conditions like asthma or anxiety, as well as other urgent events relevant to housing quality, like injuries. Using the regression discontinuity approach, I found no evidence for an effect of the housing remediation policy on ED utilization rates, measured as the probability of having one or more ED visit in the year after the AEP start: the treatment effect as measured by local linear regression was 0.28 percentage points from a baseline rate of 26% (95% CI: −3.51 to 5.19 percentage points). There was also no evidence of a change in the number of total ED visits, with an estimate of 0.73 additional visits per 100 tenants off a base of 48 visits per 100 tenants (95% CI: −6.75 to 11.34).

The next set of results limited the ED visits to conditions shown in prior literature to be associated with low-quality housing—anxiety and depression, injuries, and asthma or other chronic respiratory issues. Using a binary metric of one or more ED visits for these specific conditions, I find no evidence of an effect of housing quality improvement on acute utilization for housing-sensitive conditions (coefficient: 0.60 percentage points off a base of 18%; 95% CI: −1.60 to 5.70). A continuous count of these visits also shows no evidence of an effect (coefficient: 2.75 visits per 100 tenants off a base of 32 visits per 100 tenants; 95% CI: −3.50, 13.49). On a scale counting the number of housing-sensitive conditions tenants were seen for in any setting (inpatient or outpatient), I also found no evidence of a change in risk following the housing quality improvement (coefficient: 0.01 units off a base of 0.7 units; 95% CI: −0.05, 0.10).

Finally, as a global measure of utilization and disease burden, I tested total Medicaid expenditures of tenants in the year after AEP. This outcome also showed no evidence of a change following the housing quality improvement policy, with an effect estimate of $14.51 per year off a base of $6,661 (95% CI: −$498, $1870).

As secondary outcomes, I tested a metric of Medicaid disenrollment after the initial policy year as well as a metric capturing moves out of the building. There was no evidence of changes in either of these metrics: the effect on months enrolled in the second year after AEP start was −0.14 months (95% CI: −0.38, 0.25) off a base of 11 months, and the effect on the share who moved out of the building within a year of program start was −1.38 percentage points (95% CI: −7.46, 6.94) off a base of 22%. Neither of these effects was meaningfully large or statistically significant.

### 4.3 | Subgroup and Sensitivity Analyses

I re-ran the analyses among three particularly vulnerable groups: children, people with prior chronic respiratory or anxiety conditions, and people in buildings with at least one mold or pest violation in the year prior to AEP start. In all of these cases, the results continued to show no evidence of improvements in health utilization following the AEP intervention (Appendix Table A2 in the Supporting Information). Notably, the estimates were less precise due to the decreased sample size (and thus increased noise in the estimation of the local linear regression on either side of the cutoff from which the effect is calculated). There were also no clear patterns of impacts when stratifying the dataset by the era of AEP (2007–2009 vs, 2011–2012 vs. 2016–2018; see Appendix Table A3 in the Supporting Information): The results in these smaller samples were also much noisier with wide confidence intervals, thus making it difficult to rule out the possibility of moderate increases or decreases in utilization. Still, the fact that the effect estimates were consistently close to zero across all outcomes and across eras of the program builds the case that the housing remediation policy likely did not alter tenants’ utilization in substantial, systematic ways.

Altering the functional form from local linear regression to local quadratic regression and changing the bandwidth to 12 ranks and to 50 ranks also did not result in evidence of large changes in utilization following housing remediation (Appendix Table A4 in the Supporting Information). For some outcomes, the quadratic specification and the smaller bandwidth (12 ranks) actually showed effects that suggested small *increases* in ED utilization for buildings that experienced the housing improvement program. However, the confidence intervals of these effects still comfortably include 0 and, taken in the context of all of the other results, suggest that there is no compelling evidence of the program reducing health utilization.

To be considered compliant with AEP, a building has to correct all heat or hot water violations, as well as 80% of serious mold, pest or rodent, and other “immediately hazardous” violations (e.g., peeling lead paint, broken fixtures or windows, water leaks, etc.) within 4 months of notification. Even when limiting to enrollees in buildings that were compliant (*n* = 3662), there continued to be no evidence of an impact on acute care use (Table 2). The design of AEP also means that some buildings will move in and out of treatment status over time: some control buildings may have been previously treated or will be treated in the future. To mitigate potential bias from this, I included a sensitivity analysis using only the control buildings that were never actually selected for AEP (Table 2). These results—while consistent with the primary null findings—should be interpreted with caution. These analyses may violate the assumption that the buildings on either of side of the cutoff are equivalent, as buildings that comply with the policy are likely different in unobservable ways from those that do not. Likewise, control buildings that come close to being selected in 1 year but *manage to avoid* selection in subsequent years are likely different from control buildings that come close to selection in one year and *are* selected in subsequent years.

### 4.4 Long-Run Exploratory Analyses

Finally, I conducted exploratory analyses of long-run outcomes using two approaches (results shown in Table 3 and Appendix Figure A2 in the Supporting Information). First, I implemented the same RD approach, but testing outcomes in the second, third, and fourth years following intervention (i.e., months *T* + 13–24, *T* + 25–36, and *T* + 37–48) rather than just the first year. Unfortunately, roughly 30% of untreated buildings become treated in subsequent years, which introduces treatment spillover risks, and this specification also reduces sample size (as individuals needed to be enrolled in Medicaid for additional years). Still, some longer-run effects may be detectable with this approach. The results from this exploratory long-run RD analysis continued to show no evidence of reductions in utilization or spending; however, the coefficients for ED visits do become increasingly negative (though non-significant) with each additional year, which may weakly imply a trend toward lower ED utilization as additional years from the intervention pass.

Second, I constructed a panel dataset following individuals living in control and treated buildings at the time of AEP selection (or near-selection) for 4 years before and after the intervention. I then ran an event study analysis that took the following form:

$$y_{ibt}=\beta_{0}+\beta_{1}\;{\text{Treated}}_{ib}+\beta_{2}{\text{RelYear}}_{ibt}+\beta_{3}{\text{Treated}}_{ib}*{\text{RelYear}}_{ibt}+\delta_{it}+\gamma_{bt}+\varepsilon_{ibt}$$

where *y*_*ibt*_ is a given outcome *y* at time *t* for individual *i* who was living in building b at the time of AEP selection (or near-selection). Enrollees were not required to remain in the sample all 8 years, and I therefore controlled for individual-level and building-level characteristics that may vary over the 8-year window (*δ*_*it*_, *γ*_*bt*_) to adjust for compositional differences from period to period (age, baseline health, sex, building size, borough, calendar year, race/ethnicity). This approach gives a slightly different estimand than the RD approach, as it is the average treatment effect on the treated (ATT, or the general difference between those in buildings subjected to AEP vs. those in buildings that were under consideration for AEP but not subjected to it) rather than the LATE that is estimated by the RD (or the direct treatment effect for units most similar to each other directly at the cutoff as an emulation of a randomized trial). The event study also includes a broader range of subjects, both because we can use individuals farther from the cutoff and because the panel design can accommodate movement of individuals in and out of the sample over time. The results from this exploratory event study approach continue to show little evidence of long-run impacts to most measures of utilization or spending 4 years later (Table 3 and Appendix Figure A2 in the Supporting Information). However, there is a statistically significant decrease in the number of ED visits in the 3rd and 4th years after intervention (a 15% decrease from baseline or about 9.5 fewer visits from a baseline of 50 visits per 100 people, *p* < 0.001). Additionally, as with the long-run RD results, the acute care use coefficients generally trend more negatively with each additional year, even when non-significant. As an exploratory analysis, this implies that future work on housing quality may benefit from prioritizing program and research designs that are equipped to capture longer-run health utilization outcomes.

## 5 | Discussion and Conclusion

Low-quality housing has been correlated with a variety of physical and mental health conditions, and improving the quality of people’s living environments may benefit general wellbeing (Koops-Van Hoffen et al. 2023). Housing quality improvement interventions have been an area of growing interest for health payers like Medicaid and Medicare and other health system actors. Whether housing quality improvement policies can causally reduce acute health utilization or expenditures is not clear in the literature, yet these health sector policies and programs are often premised on the expectation of short-run returns on investment via direct impacts on health care utilization.

This quasi-experimental evaluation of the AEP—a large-scale and intensive housing remediation policy in NYC—found little evidence of changes in short-run health care utilization among tenants of buildings impacted by the policy. While rates of severe open violations in these buildings were halved within a year, I did not find evidence of meaningful or significant short-run changes in ED visit rates, visit rates for conditions believed to be sensitive to housing quality, or overall expenditures following the policy. Moderate increases of 20% or decreases of 12% in acute care use cannot be ruled out with this design and analysis, but the point estimates across specifications were consistently close to 0. In the longer run (4 years), there is some evidence that ED visit rates may be reduced by up to 10%–15%, but there is no evidence that total expenditures decrease over any time horizon tested in this analysis. This paper uses a strong causal inference design—a regression discontinuity design—that leverages the arbitrary ranking threshold used by HPD to select buildings for the program to construct an “as-good-as-random” comparison group of untreated buildings. These null results persisted across a number of sensitivity analyses and subgroup analyses, including tests among children and people with pre-existing conditions, buildings that were most compliant with the policy, and across all iterations of the policy’s ever-evolving design. The results remained null even when varying the functional form assumptions of the local polynomial regressions (e.g., linear vs. quadratic; varying sizes of bandwidths). The point estimates of the effect were consistently very close to 0, and visual inspection of plots confirms no apparent effect of the policy on utilization outcomes. The longer-run analyses showing some evidence of reduced ED visits 3–4 years later imply that future programs aiming to reduce health care utilization via housing quality improvement may consider targeting longer time horizons than current programs and policies do.

This analysis included tens of thousands of low-income residents of NYC, and the results were consistent even when the criteria for the cutoff changed over the program’s iterations over many years. This is an important contribution to the literature around housing quality policy, as the results are likely generalizable to other intensive housing quality improvement policies that target substandard buildings in other locales. However, the nature of AEP—that it only focuses on the very worst buildings—means that changes in housing quality would likely have to be even more dramatic than those AEP achieves to have a chance to influencing health spending or utilization. These buildings start in the 98th percentile of all multi-family homes in NYC terms of the number of open violations (14 open violations/unit), and even though AEP manages to halve the number of open violations within a year, the buildings are still around the 96th percentile (7 open violations/unit) the year after the remediation program begins (or the 94th percentile of buildings with at least one violation). This underscores the ongoing problem of exposure to substandard living conditions for low-income urban residents, which is relevant for policies both related and unrelated to health and wellbeing. This may be one potential explanation for the lack of significant impacts on health utilization.

Additionally, the people living in these buildings have likely lived in these same buildings—or buildings in just as dire condition—for many years. A one-time quality improvement intervention may not be sufficient to undo years of disinvestment and accumulated disadvantage experienced by these groups. Governments and nonprofit housing agencies have shown growing interest in building completely new affordable housing that is designed in line with environmental best practices, which may be a more promising route for protecting the health of low-income families in ways that smaller-scale renovations or mitigation efforts of the existing deteriorating stock of low-income housing cannot (Howden-Chapman et al. 2023; Krieger 2010; Takaro et al. 2011; U.S. Environmental Protection Agency 2018), assuming sufficient resources and political capital are available.

The evidence in this large quasi-experimental study tracks with several small randomized trials in this space, which find few meaningful improvements in utilization-based metrics of health following housing quality improvements, even when people report feeling healthier or more satisfied (Braun et al. 2018; Howden-Chapman et al. 2007; Olson et al. 2021). Policies like AEP are probably valuable in ways that cannot be measured by health care utilization data, which is an argument that has been made elsewhere (Fichtenberg and Fraze 2023; Glied 2020; Glied and D’Aunno 2023). Put differently, reducing health care expenditures should not be the primary or only motivation for investing in higher quality housing for low-income groups. However, because health payers are increasingly piloting reimbursement policies that pay for housing remediation activities like pest management with the rationale of reducing downstream utilization (and thus total costs), these results raise the question of whether this is likely to actually be true and whether this is an appropriate rationale. Indeed, several New York State Medicaid managed care plans recently announced plans to reimburse for integrated pest management at the homes of enrollees’ with asthma, with the stated goal of recouping the expense through downstream savings via lowered acute care use (Enterprise Community Partners 2022; Larson 2022), and Medicare Advantage plans have been permitted to offer supplemental benefits related to housing quality, like air filters, pest management, or accessibility modifications since 2020 (Hostetter and Klein 2020; Livingston 2019). Reframing the goals of interventions like pest management or comprehensive housing renovations to focus on the other quality-of-life improvements they may confer—rather than endorsing a narrow focus on using these interventions to bring about health care savings—may be necessary.

This finding also dovetails with recent work re-examining what factors drive issues like asthma exacerbations. Recent evidence has begun to question the relative importance of home environment over more systemic issues like ambient outdoor air pollution; unsafe worksites, schools, or offices; repeated exposure to infections agents like the cold or flu; and structural barriers to successfully managing chronic respiratory conditions, like unaccommodating employers, poor health care access, or low health literacy (Lovinsky-Desir and Volerman 2023; Zhang 2021). This quasi-experimental AEP evaluation may contribute to the discussion around this evolving understanding of the role of the home environment in shaping health care utilization.

The primary limitation of this analysis is the moderately wide confidence intervals of the estimated effects due to remaining unexplained variation in the outcomes. Utilization-based outcomes can be relatively noisy, given the many factors that can influence an individual’s risk of acute care use or health spending. While the sample is large—over 24,000 people within 25 ranks of the threshold—and I control for known correlates of utilization to increase precision, RD analyses can be underpowered as they rely heavily on the ability to accurately and precisely model the underlying function with just a few binned data points on either side of the cutoff (Cattaneo et al. 2019; Schochet 2009). I cannot necessarily rule out moderate increases or decreases in utilization from a policy like AEP due to remaining unexplained variance in the outcomes decreases (i.e., the confidence intervals include 20% relative increases and 12% relative decreases in acute care risk). Still, the fact that the point estimates of the effect are consistently close to zero across specifications suggests that the true effect is likely quite close to zero. Corroborating the findings through analyses of similar policies or by adding additional years of data as the policy continues into the future would strengthen confidence in the results.

Additionally, HPD relies in part on maintenance violation rates for determining ranks, which can be driven by tenant-initiated complaints. Buildings that appear to be “worst” per this metric may also represent buildings with the most vocal or empowered tenants (Kontokosta and Hong 2021). While this characteristic is unlikely to bias the results, as buildings on either side of the cutoff are likely to be similar in this respect, it does raise a question of generalizability and among what types of populations these results are relevant. It is possible that the treatment effect of housing quality improvements may be larger among people who are the *least* empowered, but those buildings never end up in AEP due to underreporting of violations. Tenants were typically not relocated during the remediation period; some distress or disruption from having work done on their building or unit could also offset positive effects from improved housing quality (in the short run). Finally, this study was limited to measuring claims-based utilization outcomes, which cannot fully capture tenant wellbeing. Future research should incorporate a wider range of tenant outcomes.

Cities like New York are in the throes of a major housing affordability crisis, with an estimated 500,000 units needing to be added in NYC in the next decade and the City launching numerous initiatives to build affordable housing (Office of the Mayor 2024). With the spotlight on housing affordability, efforts to ensure that urban housing—both new and old—is of adequate quality have been lower on policymakers’ priority lists. At the same time, growing interest in the health care sector in using health system dollars for housing quality improvements is raising questions of whether housing quality interventions can reduce health expenditures. Generating high-quality evidence from real-world programs can better inform these evolving cross-sector efforts, including the viability of and framing of health system investments in housing sector problems.

## Supplementary Material

Appendix

Additional supporting information can be found online in the Supporting Information section.

pam70074-sup-0001-Appendix.pdf

## Figures and Tables

**TABLE 1 |:** Local linear regression estimates of Alternative Enforcement Program (AEP) status by demographic and building characteristics.

	Coefficient	95% CI
**Individual characteristics**		
Age in years, mean	0.44	(−2.18, 4.74)
Female, percentage points	2.01	(−2.03, 5.56)
Black or Hispanic, percentage points	2.84	(−4.43, 9.79)
Baseline months on Medicaid (year prior), mean	0.01	(−0.10, 0.46)
Baseline Medicaid spending in $ (year prior), mean	−72.80	(−1865.29, 2232.40)
**Building characteristics**		
Total units, mean	6.09	(−6.66, 19.97)
Year of AEP cycle, mean	−0.01	(−1.62, 2.27)
Located in Brooklyn or the Bronx, percentage points	2.52	(−10.55, 26.60)

*Note*: Estimates come from a local linear regression with triangular kernel weights with a bandwidth of 25 ranks. The coefficient estimates the change in the variable’s mean at the AEP selection threshold (rank = 0). There are 14,974 untreated individuals and 9320 treated individuals. Outcomes are measured over the 12 months following selection into each round of AEP (2007, 2008, 2009, 2011, 2012, 2016, 2017, 2018). Standard errors are clustered by building and adjusted to account for misspecification bias.

* *p* < 0.05;

** *p* < 0.01;

*** *p* < 0.001.

**TABLE 2 |:** Local linear regression estimates of AEP treatment in the year after intervention.

	Unadjusted		Adjusted (preferred specification)		Never-treated control buildings		Compliant treated buildings	
	Effect	95% CI	Effect	95% CI	Effect	95% CI	Effect	95% CI
**Primary outcomes**								
1+ ED visit, pet pt	1.57	(−3.01, 7.07)	0.28	(−3.51, 5.19)	−0.20	(−5.47, 4.36)	0.70	(−3.54, 6.73)
Total ED visits, per 100	3.72	(−7.46,15.64)	0.73	(−6.75,11.34)	−0.89	(−15.57, 5.15)	0.45	(9.74,12.09)
1+ ED visit for housing sensitive condition, pet pt	1.72	(−0.94, 7.13)	0.60	(−1.60, 5.70)	−0.23	(−3.95, 4.96)	−0.26	(−2.83, 4.55)
Total ED visits for housing sens, conditions, per 100	5.23	(−1.56,17.17)	2.75	(−3.50,13.49)	0.31	(−11.12,12.83)	0.91	(−6.69,12.43)
Index of housing sens, visits, scale 0–3	0.01	(−0.05, 0.10)	0.01	(−0.05, 0.10)	0.01	(−0.04, 0.13)	−0.01	(−0.07, 0.08)
Total expenditures, dollars	12.48	(−1015, 2589)	14.51	(−498,1870)	−409.20	(−1513,1074)	−174.00	(−888, 1923)
**Secondary outcomes**								
Months enrolled in Medicaid in the 2nd year, mos	−0.14	(−0.41, 0.26)	−0.14	(−0.38, 0.25)	−0.20	(−0.52, 0.17)	−0.37*	(−0.87, −0.05)
Moved out of building by 12 months, pet pt	−0.68	(−6.70, 7.09)	−1.38	(−7.46, 6.94)	1.99	(−2.51,12.48)	−1.73	(−10.14, 5.85)
*N*		24,294		20,506		17,409		16,333

*Note*: Estimates come from a local linear regression with triangular kernel weights with a bandwidth of 25 ranks. The coefficient estimates the change in the variable’s mean at the AEP selection threshold (rank = 0). There are 14,974 untreated individuals and 9,320 treated individuals in the full specification. Models (except the “unadjusted” column) are adjusted for year of the AEP cycle, race/ethnicity, baseline health, age, building size, borough, and sex to increase precision. Outcomes are measured over the 12 months following selection into each round of AEP (2007, 2008, 2009, 2011, 2012, 2016, 2017, 2018). Standard errors are clustered by building and adjusted to account for misspecification bias.

* *p* < 0.05;

** *p* < 0.01;

*** *p* < 0.001.

**TABLE 3 |:** Long-run changes in utilization.

	Year 1		Year 2		Year 3		Year 4	
	Effect	95% CI	Effect	95% CI	Effect	95% CI	Effect	95% CI
**1+ ED visit, pct pt**								
*RD estimate (LATE)*	0.28	(−3.51, 5.19)	0.02	(−3.41, 4.79)	−0.31	(−4.55, 4.63)	0.42	(−4.35, 4.97)
*Event study estimate (ATT)*	−0.68	(−2.11, 0.76)	−1.23	(−2.83, 0.36)	−0.97	(−2.70, 0.76)	−1.52	(−3.40, 0.37)
**Total ED visits, per 100**								
*RD estimate (LATE)*	0.73	(−6.75, 11.34)	−0.08	(−9.66, 12.22)	−0.66	(−8.40, 15.64)	−3.48	(−13.54, 22.08)
*Event study estimate (ATT)*	−3.22	(−6.89, 1.04)	−3.98	(−8.17, 0.21)	−6.91**	(−11.69, −2.13)	−9.52**	(−16.73, −2.30)
**1+ ED (housing sens.), pct pt**								
*RD estimate (LATE)*	0.60	(−1.60, 5.70)	0.36	(−2.13, 4.74)	0.42	(−3.12, 5.20)	−0.80	(−5.67, 2.37)
*Event study estimate (ATT)*	0.39	(−0.87,1.65)	0.08	(−1.25, 1.42)	−0.47	(−1.82, 0.89)	−1.09	(−2.70, 0.52)
**Total ED (housing sens.), per 100**								
*RD estimate (LATE)*	2.75	(−3.50, 13.49)	2.01	(−6.35, 12.97)	3.92	(−4.93, 14.77)	2.50	(−6.13, 13.39)
*Event study estimate (ATT)*	0.42	(−2.45, 3.39)	−1.22	(−4.66, 2.23)	−1.62	(−5.13, 1.89)	−2.73	(−7.00, 1.53)
**Index of housing sens. visits, 0**–**3**								
*RD estimate (LATE)*	0.01	(−0.05, 0.10)	0.02	(−0.05, 0.11)	−0.01	(−0.12, 0.06)	−0.01	(−0.7, 0.11)
*Event study estimate (ATT)*	0.01	(−0.01, 0.02)	−0.00	(−0.02, 0.02)	−0.01	(−0.02, 0.01)	−0.01	(−0.03, 0.01)
**Total expenditures, dollars**								
*RD estimate (LATE)*	14.51	(−498, 1870)	−305.96	(−1603, 1451)	−155.88	(−1650, 2119)	−280.00	(−2340, 1511)
*Event study estimate (ATT)*	246.09	(−225, 717)	625.98	(−202, 1454)	512.37	(−344, 1369)	−51.69	(−993, 890)
**N**								
*RD estimate (LATE)*		20,506		18,943		15,361		12,364
*Event study estimate (ATT)*		24,294		23,325		19,535		16,129

*Note*: Estimates for the RD specifications come from a local linear regression with triangular kernel weights with a bandwidth of 25 ranks. The coefficient estimates the change in the variable’s mean at the AEP selection threshold (rank = 0). Estimates for the Event Study specification come from a model interacting relative year fixed effects with a treatment indicator fixed effect (see Results text for details). RD models are adjusted for year of the AEP cycle, race/ethnicity, baseline health, age, building size, borough, and sex to increase precision. Event study models are adjusted for calendar year, race/ethnicity, building size, borough, and sex to account for compositional differences in who is observed over time. Outcomes are measured over 12-month periods following selection into each round of AEP (2007, 2008, 2009, 2011, 2012, 2016, 2017, 2018)—for example, Year 1 is the 12 months directly after intervention, while Year 2 is months 13–24. Standard errors are robust and clustered by building.

* *p* < 0.05;

** *p* < 0.01;

*** *p* < 0.001.

## Data Availability

The Medicaid claims and encounters data used in this analysis are confidential and contain protected health information. They cannot be shared publicly. Those interested in accessing the data should arrange a data use agreement with the New York State Department of Health. Alternative Enforcement Program ranking data are public but must be requested through a FOIL request.
