# Supplementary material for: Housing Quality Improvement and Health Care Utilization: A Regression Discontinuity Study
Source: J Policy Anal Manage. Author manuscript; Available in PMC 2026 Apr 16. (PMC13082781; doi:10.1002/pam.70074)
Supplement: Appendix [file NIHMS2162953-supplement-Appendix.pdf]

APPENDIX FOR:

Housing Quality Improvement and Health Care Utilization: A Regression Discontinuity Study

Appendix Table A1: AEP Policy Rules over Time

**Year**	**Initial Screening Criteria** (All buildings under consideration in all rounds must have 3+ units and not be part of the public housing system, as well as the year-specific criteria below)	**Ranking variable** (variable that determines cutoff)	**Total buildings chosen**	**Note**
2007-2008	27+ total open class B or C violations in the past 2 years AND a ratio of 5 or more open class B and C violations per unit in the past 2 years AND unpaid emergency repair charges of $100 per unit or more in the past 2 years	Ratio of class B & C violations per unit	200	
2009	25+ total open class B or C violations in the past 2 years AND a ratio of 5 or more open class B and C violations per unit in the past 2 years AND paid or unpaid emergency repair charges of $100 per unit or more in the past 2 years	Ratio of class B & C violations per unit	200	
2011-2012	For buildings >= 20 units: A ratio of 3 or more open class B and C violations per unit in the past 2 years AND paid or unpaid emergency repair charges of $5000 total or more in the past 2 years<br><br>For buildings <20 units: A ratio of 5 or more open class B and C violations per unit in the past 2 years AND paid or unpaid emergency repair charges of $2500 total or more in the past 2 years	Total paid or unpaid emergency repair charges in the past 2 years	200	

2013-2014	For buildings >= 20 units:<br>A ratio of 3 or more open class B and C violations per unit in the past 3 years<br>AND paid or unpaid emergency repair charges of $2500 total or more in the past 3 years<br><br>For buildings <20 units:<br>A ratio of 5 or more open class B and C violations per unit in the past 3 years<br>AND paid or unpaid emergency repair charges of $5000 total or more in the past 3 years	Total paid or unpaid emergency repair charges in the past 2 years	200	Excluded from study due to unavailable ranking data
2015	For buildings >= 20 units:<br>A ratio of 3 or more open class B and C violations per unit in the past 5 years<br>AND paid or unpaid emergency repair charges of $2500 total or more in the past 5 years<br><br>For buildings <20 units:<br>A ratio of 5 or more open class B and C violations per unit in the past 5 years<br>AND paid or unpaid emergency repair charges of $5000 total or more in the past 5 years	Total paid or unpaid emergency repair charges in the past 2 years	250	Excluded from study due to unavailable ranking data
2016-2018	Building size >=6 units<br>AND a ratio of 4 or more open class B or C violations per unity in the past 5 years	Total class B or C violations in the past 5 years	250	There are two sets of criteria for these years, but the second set is the relevant set for buildings at the cutoff (the other set largely pertains to small buildings which are at the top of the list per the criteria)

Appendix Figure A1: Covariate smoothness at cutoff: year of program cycle and pre-intervention months enrolled

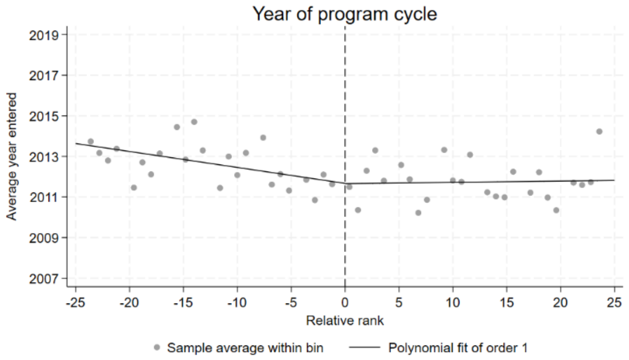

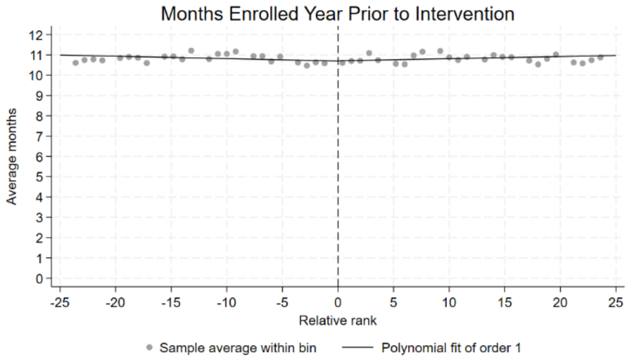

Appendix Table A2: Subgroup analyses

	Children		People with Prior Chronic Conditions		Buildings with Mold/Pests	
	**Coefficient (local average treatment effect)**	**95% CI**	**Coefficient (local average treatment effect)**	**95% CI**	**Coefficient (local average treatment effect)**	**95% CI**
**Primary outcomes**						
1+ ED visit, pct pt	0.60	(-4.32, 7.49)	0.58	(-4.78, 7.34)	-1.78	(-6.83, 4.12)
Total ED visits, per 100	4.10	(-4.09, 19.23)	2.66	(-9.48, 20.33)	-2.26	(-13.50, 8.05)
1+ ED visit for housing sensitive condition, pct pt	-0.77	(-4.82, 5.37)	0.50	(-3.26, 6.75)	-0.13	(-2.93, 5.26)
Total ED visits for housing sens. conditions, per 100	2.32	(-5.27, 12.20)	3.33	(-11.02, 21.40)	1.53	(-8.51, 12.56)
Index of housing sens. visits, scale 0-3	-0.00	(-0.09, 0.11)	0.01	(-0.08, 0.12)	0.01	(-0.06, 0.10)
Total expenditures, dollars	-32.42	(-890, 1597)	737.54	(-237, 3534)	426.68	(-440, 2755)
**Secondary outcomes**						
Months enrolled in Medicaid in the 2nd year, mos	-0.22	(-0.52, 0.14)	0.06	(-0.28, 0.35)	-0.11	(-0.49, 0.33)
Moved out of building by 12 months, pct pt	-1.50	(-9.44, 8.86)	-1.97	(-9.59, 7.13)	-3.37	(-12.85, 3.84)
N		11,303		10,246		16,393

*p<0.05, **p<0.01, ***p<0.001.

Appendix Table A3: Analyses by era of the policy

	2007-2009		2011-2012		2016-2018	
	**Coefficient (local average treatment effect)**	**95% CI**	**Coefficient (local average treatment effect)**	**95% CI**	**Coefficient (local average treatment effect)**	**95% CI**
**Primary outcomes**						
1+ ED visit, pct pt	0.62	(-5.68, 7.21)	3.88	(-3.38, 15.11)	-1.45	(-6.94, 7.61)
Total ED visits, per 100	1.23	(-7.58, 13.49)	7.58	(-8.36, 31.75)	-2.58	(-17.35, 19.33)
1+ ED visit for housing sensitive condition, pct pt	2.15	(-1.31, 8.63)	1.48	(-6.50, 12.26)	-1.78	(-5.92, 5.80)
Total ED visits for housing sens. conditions, per 100	4.09	(-0.80, 13.53)	12.20	(-0.96, 37.64)	-4.70	(-22.42, 15.03)
Index of housing sens. visits, scale 0-3	-0.01	(-0.10, 0.12)	0.06	(-0.14, 0.16)	0.03	(-0.04, 0.18)
Total expenditures, dollars	-311.20	(-1608, 1457)	22.37	(-2891, 2392)	91.42	(-825, 3347)
**Secondary outcomes**						
Months enrolled in Medicaid in the 2nd year, mos	-0.16	(-0.46, 0.31)	-0.47	(-1.53, 0.13)	0.06	(-0.21, 0.80)
Moved out of building by 12 months, pct pt	-2.94	(-12.44, 11.46)	4.26	(-5.78, 25.72)	-0.93	(-10.99, 7.01)
N		9,987		4,635		9,672

*p<0.05, **p<0.01, ***p<0.001.

Appendix Table A4: Analyses by various bandwidth and functional form choices

	Quadratic, bandwidth 25		Linear, bandwidth 12		Linear, bandwidth 50	
	**Coefficient (local average treatment effect)**	**95% CI**	**Coefficient (local average treatment effect)**	**95% CI**	**Coefficient (local average treatment effect)**	**95% CI**
**Primary outcomes**						
1+ ED visit, pct pt	0.92	(-3.00, 8.67)	1.45	(-3.00, 9.96)	0.10	(-2.88, 3.86)
Total ED visits, per 100	2.70	(-8.86, 14.36)	0.70	(-3.46, 23.20)	-1.20	(-6.35, 7.97)
1+ ED visit for housing sensitive condition, pct pt	2.14	(-0.68, 9.17)	2.49	(-0.75, 10.39)	0.57	(-2.06, 3.41)
Total ED visits for housing sens. conditions, per 100	5.11	(-1.52, 20.29)	5.34*	(0.56, 24.33)	1.58	(-4.28, 8.71)
Index of housing sens. visits, scale 0-3	0.02	(-0.02, 0.17)	0.04	(-0.03, 0.17)	0.01	(-0.04, 0.07)
Total expenditures, dollars	722.60	(-1314, 1836)	510.31	(-1449, 1602)	-294.20	(-994, 737)
**Secondary outcomes**						
Months enrolled in Medicaid in the 2nd year, mos	-0.06	(-0.33, 0.51)	-0.06	(-0.26, 0.66)	0.05	(-0.38, 0.10)
Moved out of building by 12 months, pct pt	-0.11	(-9.71, 8.79)	0.65	(-12.06, 7.46)	-0.94	(-6.90, 3.98)

*p<0.05, **p<0.01, ***p<0.001.

Figure A2: Event study plots for up to 4 years before and after intervention.

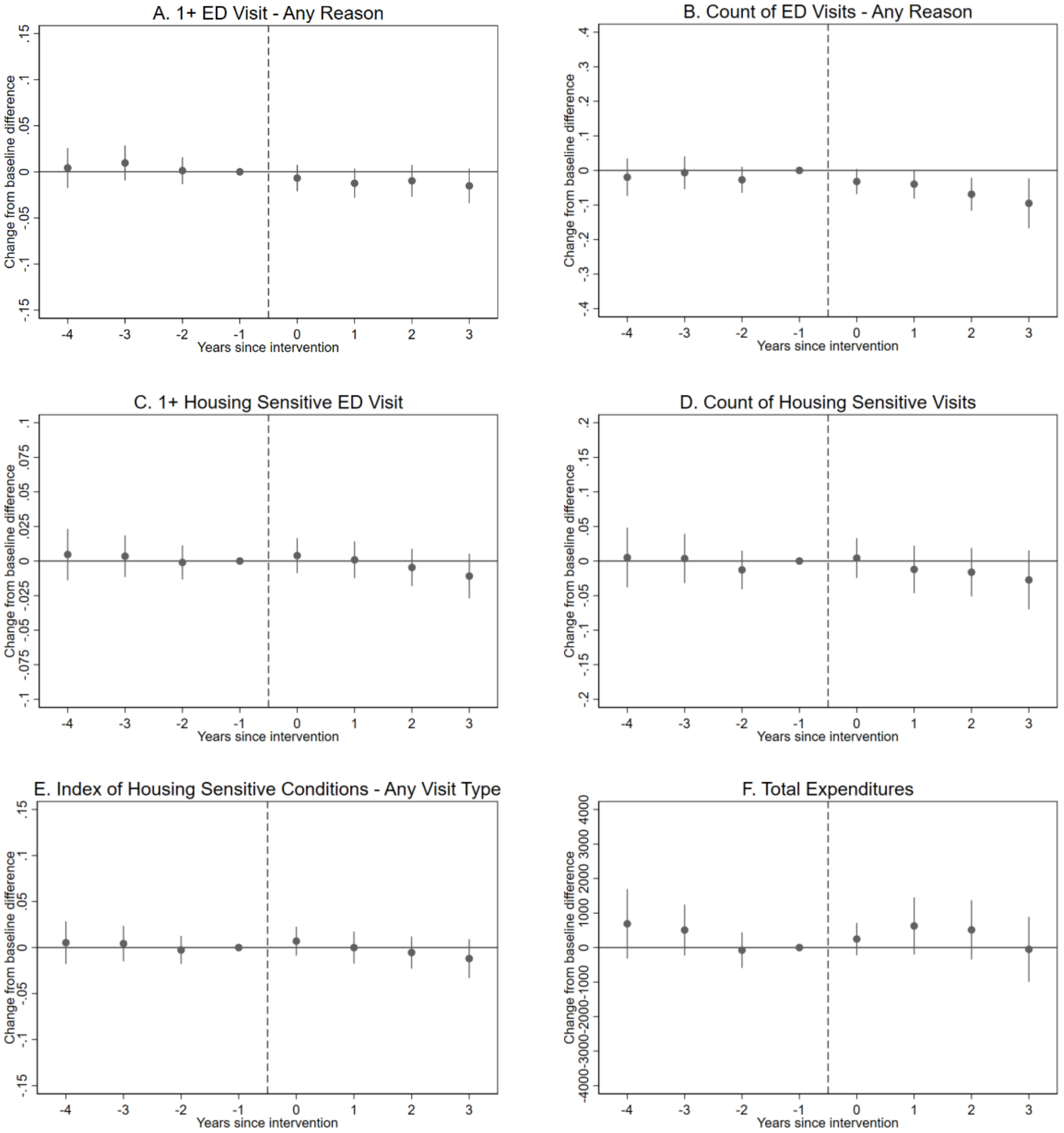

Notes: Each panel shows the event study estimates from a model interacting relative year fixed effects with a treatment indicator fixed effect (see Results section of manuscript for detailed methods), which represents the additional change in each year relative to baseline differences between the treated and control groups. Models are adjusted for calendar year, race/ethnicity, baseline health, age, building size, borough, and sex to account for compositional differences in the observed enrollees over time. Outcomes are measured over 12 month-periods following selection (or, near-selection) into each round of AEP (2007, 2008, 2009, 2011, 2012, 2016, 2017, 2018) – for example year 1 is the 12 months directly after intervention, while year 2 is months 13-24. Standard errors are robust and clustered by building.
